# The impact of *Lactobacillus acidophilus* on hepatic and colonic fibrosis induced by ethephon in a rat model

**DOI:** 10.22038/ijbms.2019.32936.7866

**Published:** 2019-08

**Authors:** Hoda I Bahr, Rania Hamad, Shimaa AA Ismail

**Affiliations:** 1 Biochemistry Department, Faculty of Veterinary Medicine, Suez Canal University, Ismailia 41522, Egypt; 2 Pathology Department, Faculty of Veterinary Medicine, Suez Canal University, Ismailia 41522, Egypt; 3 Clinical Pathology Department, Faculty of Veterinary Medicine, Zagazig University, Egypt

**Keywords:** Ethephon, Immunity, L. acidophilus, NF-κB, α-SMA

## Abstract

**Objective(s):**

The study is aimed to elucidate the impact of antioxidant, anti-inflammatory and antifibrosis properties of *Lactobacillus acidophilus (L. acidophilus)* on liver and colon in ethephon treated rats through measuring Pro- inflammatory cytokines, oxidative stress index, lysosomal cathepsin-D enzyme activity and fibrosis markers.

**Materials and Methods:**

Rats divided into three groups; Group 1: distilled water control, Group 2: rats at day 16 from experiment beginning were orally received ethephon 50 mg/ kg BW in distilled water once daily for 60 days. Group 3: rats were orally received *L. acidophilus* enriched diet 1% (w/w) for 15 days as prophylactic, then received both *L. acidophilus* enriched diet 1% (w/w) and ethephon 50 mg/kg BW for 60 days.

**Results:**

Ethephon exerts hepatic and colonic oxidative stress, inflammatory response and fibrosis through NF-κB activation. On contrary, *L. acidophilus* supplementation evokes hepatoprotective properties as revealed by decreased serum AST, ALT, γ -GT and increased IGF-1. *L.* acidophilus exerts antioxidant and anti-inflammatory properties as indicated by decreased TOS, OSI, TNF-α, IL-1β, cathepsins D activity, NF-κB expression and increased TAC, lysosomal membrane stability. *L. acidophilus* shows antifibrotic activity as demonstrated by down-regulation of TGF-β1, α-SMA, collagen expression.

**Conclusion:**

*L. acidophilus* possess antioxidant, anti -inflammatory and antifibrotic activity through inhibition of NF-kB.

## Introduction

Hepatic and colonic fibrosis is the wound response to acute or chronic injury and characterized by excessive production of collagen (1, 2). Ethephon [2-Chloroethyle-phosphonic acid (C_2_H_6_ClO_3_P)] is organophosphorus compound widely used at low doses for pre-harvest ripening in mango, pineapple, coffee, tomato, cucumber, groundnut and used as herbicide at high doses (3, 4). Consumption of ethephon-treated fruits and vegetables may lead to liver, kidney diseases, cardiac disturbances, central nervous system depression, skin and gastrointestinal irritation specially in children (5-7). Previous investigations about ethephon recorded oxidative stress and reproductive toxicity in albino rat (8), mutagenic influence in albino mice (9) and hematological toxic effect in rats (10). Previous study investigated the involvement of gut-liver axis in fibrosis pathogenesis (11). Lactic acid bacteria (LAB) are vital for humans and animals (12). Many studies demonstrate antioxidant and anti-inflammatory properties of probiotic (13) reported antidiabetic impacts of probiotic dahi containing *Lactobacillus acidophilus* and *Lactobacillus casei* in rats fed on high fructose. Additionally, *L. acidophilus* and/or prebiotic inulin inhibit intestinal NF-κB and Smad 7 signaling versus exposure to *Citrobacter rodentium* (14). *L. acidophilus* R0052 and *L. rhamnosus* R001 down-regulated toll-like receptor 4 expression in alcohol- induced liver disease in mice (15). Since studies on ethephon is still limited, although it represents a hazard. Thereby, this work aimed to investigate the impact of antioxidant, anti-inflammatory and antifibrosis properties of *L.* *acidophilus* on liver and colon in ethephon treated rats through measuring Pro- inflammatory cytokines, oxidative stress index, lysosomal cathepsin-D enzyme activity and fibrosis markers.

## Materials and Methods

Ethephon (Ethrel) supplied by Bayer Crop Science, Egypt. 

Probiotic (Lacteol forte). Sachet form: *L. acidophilus*, killed and lyophilized bacteria, 10 billion (10^10^ cfu), Spent culture medium 160 mg was obtained from Rameda-pharmaceuticals Company, Egypt. 

***Animals and experimental approach***

Male Sprague–Dawley rats weighing 150-180 g purchased from Animal House in Faculty of Vet. Medicine, Zagazig University, Egypt. They were fed standard balanced ration. Feed and water supplied *ad libitum* and kept under appropriate conditions of housing and handling. The rats acclimatized to the laboratory conditions for two weeks. All rats were treated in accordance with the guideline for care and use of animals which approved by Research Ethics Committee in Faculty of Vet. Medicine, Suez Canal University.

***Experimental grouping***

Twenty-four rats randomly allocated to three groups of eight rats each as follow: 

Group 1: rats were served as a negative control, then, at day 16 from the experiment beginning orally received distilled water 5 ml/kg BW once daily for 60 days. 

Group 2: rats at day 16 from the experiment beginning were kept as a positive control and orally received ethephon 50 mg/kg BW per-os in distilled water once daily (16) for 60 days. 

Group 3: rats were orally received *L. acidophilus* enriched diet 1% (w/w) (17) for 15 days as prophylactic. Then at day 16, rats were received both ethephon 50 mg/kg BW/day per-os in distilled water and *L. acidophilus* enriched diet 1% (w/w) for 60 days. Whereas treatment with *L. acidophilus* was launched at day 1 and continued until 75 days in this group. 

At the end of the experimental period, blood samples were collected from the inferior vena cava of each rat and serum was separated for biochemical analysis. Rats were sacrificed by cervical decapitation. Part of liver and colon was used for determination of biochemical markers. Another part was used for histopathological investigation and immunohistochemistry staining.

***Serum biochemical analysis***

Serum AST, ALT and γ-GT activity was analyzed according to the manufacturer’s instructions (Biodiagnostic, Egypt). Pro-inflammatory cytokines (TNF-α, IL-1β) and IGF-1 were estimated by aid of ELISA kit of BD Pharmingen, San Diego, California, USA. TGF-β1 was estimated by aid of ELISA using TGF-B1 ELISA of Kamiya Biomedical Company, USA.

***Tissue biochemical assay***

*Assessment of oxidant/antioxidant status*

Liver and colon total antioxidant capacity (TAC) was carried following the manufacturer’s instructions (Biodiagnostic, Egypt). Total oxidant status (TOS) was analyzed using the methods described by Erel (18). Oxidative stress index (OSI) was calculated as follows: OSI (arbitrary unit)=[TOS (μmol H_2_O_2_ equivalent/g tissue)/TAC (mM H_2_O_2_ equivalent/g tissue) × 100]. 

***Determination of lysosomal cathepsin-D enzyme activity***

Total, free cathepsin-D activity and lysosomal membrane integrity estimation were determined (19, 20). The ratio of total activity/free activity is taken as the index of lysosomal membrane integrity. Enzyme activity in all cases considered as μg-released tyrosine/mg substrate protein. Total protein was determined (21).

***Histopathological examination***

Liver and colon specimen were processed and stained with H&E and Masson’s trichrome (22, 23). Collagen tissue score were analyzed using image J 1.51 p software (magnification ×40).

***Immunohistochemistry and image analysis***

Liver and colon sections were stained (24), then incubated with primary rabbit polyclonal-NF-κB p65 antibody (1: 100) against NF-κB/p65 and mouse monoclonal α-SMA antibody (1:800) against α-SMA (Thermo fisher scientific, USA) for 30 min at room temperature. Markers were visualized using biotin-streptavidin system (25). Diaminobenzidine used as a chromogen. Slides were counter stained by hematoxylin and examined using Zeiss Axioplan microscope (Carl Zeiss Microimaging, Thornwood, NY). NF-κB/p65 and α-SMA positive cells were analyzed using image J 1. 51 p software (magnification ×40). 

***Statistical analysis***

Data performed using SPSS version 22 for Windows and expressed as mean± SEM. and statistical analysis done using one-way analysis of variance (ANOVA) followed by the Duncan analysis to assess significant differences among groups. The criterion for statistical significance was set at *P*<0.05.

## Results

***Effect of ethephon and L. acidophilus treatments on some serum biomarkers***

Ethephon exerted elevation (*P*<0.05) in γ-GT, AST, ALT activities and TNF-α, IL-1b, TGF-β1 levels with reduction in IGF-1 level comparing to control group. *L. acidophilus* treatment imparts hepatoprotective and anti-inflammatory properties and able to restore these markers to normal values (Table 1). 

**Figure1:**
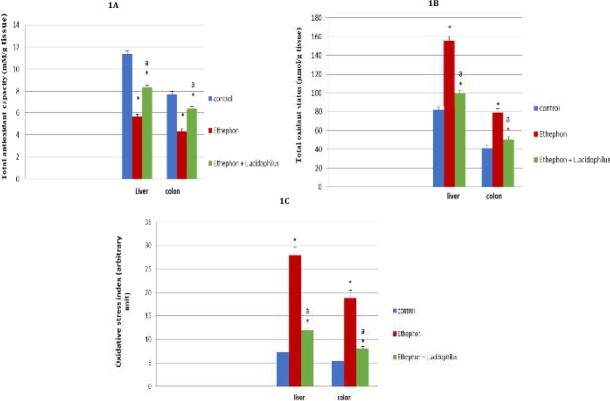
Effect of *Lactobacillus acidophilus* enriched diet supplementation on liver, colon oxidant/antioxidant status in ethephon treated rats

A) Total antioxidant capacity. B) Total oxidant status. C) Oxidative stress index. Data are expressed as mean±SEMand analyzed using one-way ANOVA followed by the Duncan analysis at *P<*0.05. ^*^P compared to control, ^a^*P* compared to ethephon- treated group

**Figure 2:**
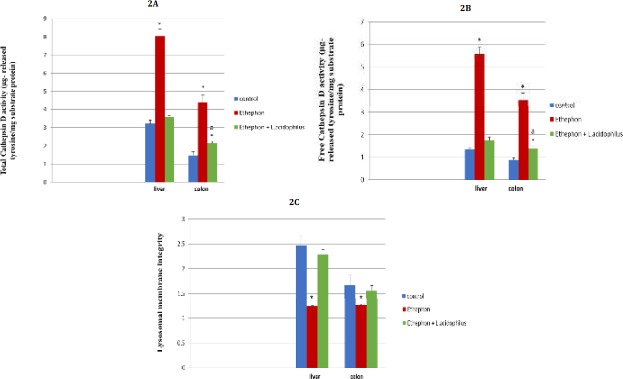
Effect of *Lactobacillus acidophilus* enriched diet supplementation on liver, colon lysosomal cathepsin D in ethephon treated rats

A) Total cathepsin D activity. B) Free cathepsin D activity. C) Lysosomal membrane integrity. Data are expressed as mean±SEM and analyzed using one-way ANOVA followed by the Duncan analysis at *P<*0.05. ^*^P compared to control, ^a^*P* compared to ethephon- treated group

**Figure 3:**
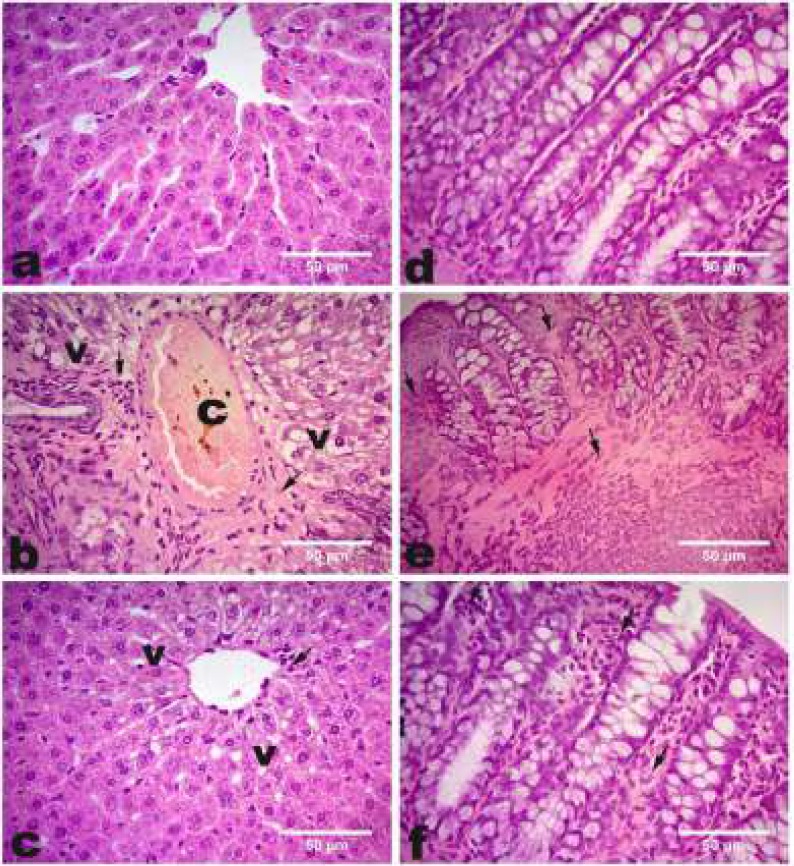
Photomicrographs showing the effect of *Lactobacillus acidophilus* enriched diet supplementation on liver, colon histopathological picture in ethephon treated rats

A–F: Representative hematoxylin and eosin staining sections of the liver and colon, (a) liver of control group (b) liver of ethephon treated group showed diffuse hepatic vacuolation (V), congestion of central vein (C) with severe fibroblast proliferation and lymphocytic infiltration (arrows)(c) liver of ethephon+ *L. acidophilus* group showed normal hepatocytes with mild vacuolation (V) and lymphocytic infiltration (arrow) (d) colon of control rats (e) colon of ethephon treated group showed degeneration of crypts of Lieberkühn with severe mononuclear cell infiltrations and severe proliferation of fibroblasts (arrow) (f) colon of ethephon+*L. acidophilus* group showed mild cellular infiltration and proliferation of fibroblast (arrow). (H&Ex40)

**Figure 4:**
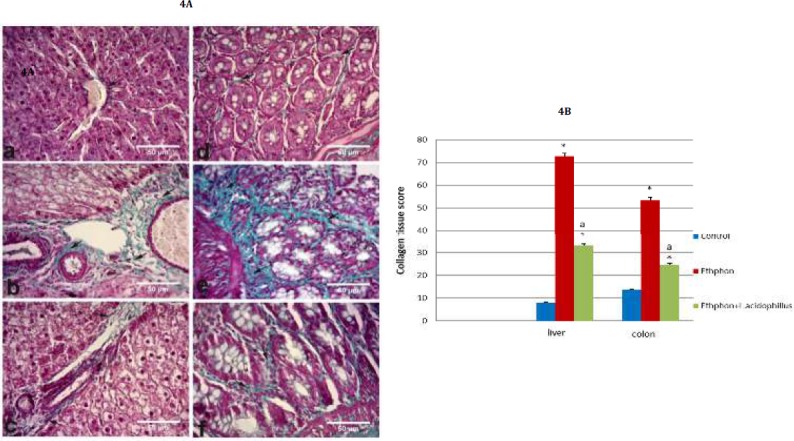
A) Photomicrographs showing Masson’s Trichrome for liver and colon; (a) liver of control group (b) Liver of ethephon group (c) liver of ethephon+*L. acidophilus* group (d) colon of control group (e) colon of ethephon group (f) colon of ethephon+*L. acidophilus* group X40. B) Effect of *L. acidophilus* enriched diet supplementation on liver, colon collagen score in ethephon treated rats. Data are expressed as mean±SEM and analyzed using one-way ANOVA followed by the Duncan analysis at *P<* 0.05. ^*^P compared to control, ^a^*P* compared to ethephon- treated group

**Figure 5:**
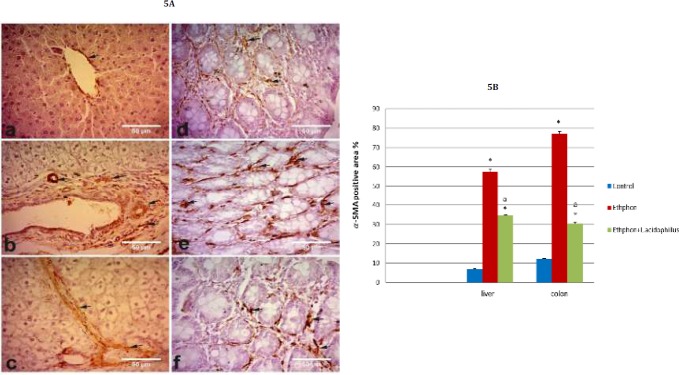
A) Photomicrographs showing immunostaining for α-SMA expression in liver and colon; (a) liver of control group (b) liver of ethephon group (c) liver of ethephon+*L. acidophilus* (d) colon of control rats (e) colon of ethephon treated group (f) colon of ethephon+*L. acidophilus* group. Arrows refer to α-SMA expression X40. B) Effect of *L. acidophilus* enriched diet supplementation on liver, colon α-SMA expression (area %) in ethephon treated rats. Data are represented mean±SEM and analyzed using one-way ANOVA followed by the Duncan analysis at *P<*0.05. ^*^P compared to control, ^a^*P* compared to ethephon- treated group

**Figure 6:**
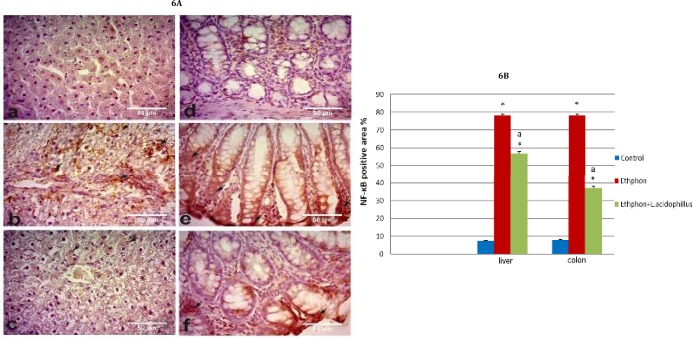
A) Photomicrographs showing immunostaining for NF-κB/ p65 expression in liver and colon; (a) liver of control group, (b) Liver of ethephon group, (c) Liver of ethephon+*L. acidophilus* group, (d) Colon of control rats, (e) colon of ethephon group, (f) Colon of ethephon+*L. acidophilus* group. Arrows refer to NF-κB/ p65 expression X40. B) Effect of *L. acidophilus* enriched diet supplementation on liver, colon NF-κB/ p65 expression (area %) in ethephon treated rats. Data are represented mean±SEM and analyzed using one-way ANOVA followed by the Duncan analysis at *P<*0.05. ^*^P compared to control, ^a^*P* compared to ethephon- treated group

**Table 1:** Effect of L. acidophilus enriched diet supplementation on serum liver enzymes activities, Pro- inflammatory cytokines and insulin-like growth factor- 1 levels in ethephon treated rats

Parameters	Groups		
	Control	Ethephon	Ethephon+ L. acidophilus
ALT (units/ml)	52.13± 1.68	92 ± 2.29*	71.25 ± 1.69 ≠
AST (units/ml)	61.19± 1.64	101.38 ± 2.28*	85.88 ± 2.43 ≠
γ -GT(U/L)	42.62± 2.41	98 ± 3.23*	74.88 ± 3.35 ≠
TNF-α (Pg/ml)	39.87± 1.78	82.63± 2.84*	61.62 ± 1.98 ≠
IL-1β (Pg/ml)	30.87± 1.44	73.87± 2.63*	46.75 ± 2.15 ≠
TGF-β1(Pg/ml)	15.31± 0.24	23.92± 0.45*	18.49 ± 0.34 ≠
IGF-1(Pg/ml)	155.63± 3.01	102.50± 3.48*	133.13 ± 2.44 ≠

Values are expressed as mean ± S.E.M. and analyzed using one-way ANOVA followed by the Duncan analysis. Data having different superscript are significant at *P*< 0.05.

* *P* Compared to control group,

≠ *P* Compared to ethephon group.

***Effect of ethephon and L. acidophilus treatments on liver and colon oxidant/antioxidant parameters***

Ethephon significantly *P*<0.05 decreased TAC along with significant increase in TOS, OSI comparing to control group. *L. acidophilus* administration evoked antioxidant properties (Figure 1). 

***Effect of ethephon and L. acidophilus treatments on liver and colon lysosomal cathepsin-D enzyme activity***

Ethephon induced marked elevation in cathepsin D (free, total) activities along with reduction in lysosomal membrane stability. These alterations alleviated toward normal by oral *L. acidophilus* administration (Figure 2). 

***Histopathological Results***

Ethephon treatment induced hepatic toxicity as indicated by diffuse hepatic vacuolation, central vein congestion, severe fibroblast proliferation and lymphocytic infiltration (Figure 3B) comparing to control (Figure 3A). While, *L. acidophilus *supplementation improved hepatocytes architecture (Figure 3C). Regarding colon, ethephon induced degeneration of crypts of Lieberkühn with loss of goblet cells, severe mononuclear cell infiltrations and severe proliferation of fibroblasts (Figure 3E) comparing to control (Figure 3D). In contrast, colonic architecture is preserved by *L. acidophilus* (Figure 3F).

Ethephon exerted severe fibroblasts proliferation comparing to control (Figure 4A) and collagen deposition in liver and colon (Figure 4B). While, *L. acidophilus* administration revealed antifibrotic properties.

***α*** ***-*** ***SMA and NF*** ***-*** ***κB*** ***/ p65 immunostaining***

Ethephon up-regulated hepatic and colonic α-SMA expression. In contrast *L. acidophilus* supplementation down-regulated α-SMA expression (Figure 5A, 5B).

Ethephon induced hepatic and colonic NF-κB/ p65 overexpression, particularly in the nuclei of macrophages and epithelial cells**.** *L. acidophilus* treatment down-regulated NF-κB/ p65 expression (Figure 6A, 6B).

## Discussion

Our data revealed that ethephon induced hepatic and colonic toxicity, oxidative stress and inflammatory response as indicated by increased serum liver enzyme leakage, TNF-α, IL-1β and TGF-β1. As well as, ethephon reduced serum IGF-1 levels that agree with the finding of Conchillo *et al.* who reported marked reduction in the levels of IGF-I in liver cirrhosis (26). Additionally, reduction in hepatic and colonic TAC and increased TOS and OSI are reported that may attribute to free radical production by ethephon (27). ROS can oxidize sulfhydryl group of antioxidants and/ or affecting hepatic antioxidant expression (28). Moreover, ethephon elevated cathepsin D (free, total) activities with reduction in lysosomal membrane integrity that may relate to lysosomal membrane damage by free radicals. This causes membrane instability and cathepsins leakage. 

Previous data implied the link between oxidative stress, inflammation and fibrosis (29). That confirmed with our findings that reveal overexpression of hepatic and colonic collagen, α-SMA and NF-κB/ p65 in ethephon treated rats. Additionally, ROS can stimulate NF-κB that induced expression of IL-6 TGF- β, and COX-2 in CCl_4_ treated- rats (30). Moreover, TNF-α and IL-1b expression are closely related to NF-kB as transcription factor (31). Interestingly, oxidative stress and cytokines can activate hepatic stellate cells (HSCs) that express α-SMA and collagen (32, 33). Hence, in accordance with our findings Liu *et al.* (34) reported lower cathepsins B and cathepsins D levels in quiescent HSCs while higher values are detected with α-SMA and TGF-β1 overproduction during HSC activation that confirming our results. Collectively, ethephon induced hepatic and colonic toxicity through NF-κB activation.

On contrary, *L. acidophilus *administration induced hepatoprotective properties as expressed by restoring liver function enzyme and IGF-1 to normal values. These results may relate to antioxidant and anti-inflammatory properties of *L. acidophilus *that confirmed by decreased TOS, OSI, TNF-α and IL-1β levels. As well as, increased TAC is associated with reduced cathepsins D activity and restored lysosomal membrane stability. Our results agree with many studies that highlighted the antioxidant and anti-inflammatory activities of *L. acidophilus***. **It down-regulates leukotriene B4, iNOS production and MPO activity in TNBS model of rat colitis (35) and down-regulated COX-2 expression in catla thymus macrophages (36). Also, Supplementation of yogurt containing *L. acidophilus* La5 and *Bifidobacterium lactis* Bb12 increased erythrocyte GSH-Px, SOD activities, total antioxidant status in diabetic patients (37). Additionally, *L. acidophilus* decreased TNF-α, IFN-γ in weaned piglets challenged with *Escherichia coli* LPS (38). Moreover, our data showed antifibrotic activity of* L. acidophilus *as indicated by decreased serum TGF-β1 level and collagen, α-SMA and NF-κB/ p65 expression. These findings are confirmed with researchers (39) who noted that VSL#3 Probiotic containing *L. acidophilus* inhibited collagen expression and TGF β1 in mice fed on methionine choline deficient diet induced liver fibrosis. Moreover, *L. acidophilus *inhibited TRL4 and NF-κB expression in peripheral blood mononuclear cells after LPS challenge (40). Many studies revealed the antioxidant efficacy of *L. acidophilus *comparing to other products. Soluble polysaccharide fraction from *L. acidophilus* 606 may consider a novel anticancer and antioxidant agent (41). Probiotic (*L. acidophilus, L. casei*, *Bifidobacterium bifidum*) induced hypoglycemic and hypolipidemic effect better than placebo in pregnant diabetic women (42). *L. acidophilus* alone is more efficient hepatoprotective than inulin or in conjunction with inulin in a murine model of *Salmonella typhimurium* caused liver damage (43)**.*** L. acidophilus* ameliorated reproductive organs oxidative stress in arthritis rat model comparing to NSAIDs (44)**.** Comparing to other probiotics, *L. acidophilus* strain is better than other bacterial strains in reduction of TC and LDL-C levels (45)**.** Recently, *L. casei/acidophilus* possess the highest antioxidant potential among other strains (46)**. **Collectively,* L. acidophilus* can possess antioxidant, anti -inflammatory and antifibrotic activity through inhibition of NF-kB.

## Conclusion

The current work highlighted that, oral consumption of *L. acidophilus* ameliorated ethephon-induced liver and colon fibrosis as indicated by down-regulation of TGF-β1, α-SMA, collagen expression through inhibition of NF-kB. Hence, *L. acidophilus* can be used a promising candidate against fibrosis.

## Financial support

This research did not receive any specific grant from funding agencies in the public, commercial, or not-for-profit sectors.
